# Hofmeister Effect Promoted the Introduction of Tunable Large Mesopores in MOFs at Low Temperature for Femtomolar ALP Detection

**DOI:** 10.1002/advs.202305786

**Published:** 2023-11-30

**Authors:** Jian Yang, Ming Gong, Fan Xia, Yao Tong, Jinlou Gu

**Affiliations:** ^1^ Key Laboratory for Ultrafine Materials of Ministry of Education School of Materials Science and Engineering East China University of Science and Technology Shanghai 200237 China

**Keywords:** femtomolar alkaline phosphatase detection, hofmeister effect, low temperature synthesis, metal‐organic frameworks, tunable large mesopore

## Abstract

In addressing the demand for hierarchically mesoporous metal‐organic frameworks (HMMOFs) with adjustable large mesopores, a method based on the synergistic effects of low‐temperature microemulsions and Hofmeister ions is developed. Low temperature dramatically enhanced the solubility of hydrophobic solvent in the microemulsion core, enlarging the mesopores in HMMOFs replica. Meanwhile, Hofmeister salt‐in ions continuously controlled mesopore expansion by modulating the permeability of swelling agent into the microemulsion core. The large mesopores up to 33 nm provided sufficient space for the alkaline phosphatase (ALP) enrichment, and retained the remaining channel to facilitate the free mass diffusion. Leveraging these advantages, a colorimetric sensor is successfully developed using large‐mesopore HMMOFs for femtomolar ALP detection based on the enrichment and cycling amplification principles. The sensor exhibited a linear detection range of 100 to 7500 fm and a limit of detection of 42 fm, presenting over 4000 times higher sensitivity than classic para‐nitrophenyl phosphate colorimetric methods. Such high sensitivity highlights the importance of adjustable mesoporous structures of HMMOFs in advanced sensing applications, and prefigures their potential for detecting large biomolecules in diagnostics and biomedical research.

## Graphical abstract

Based on the synergistic effects of low‐temperature microemulsions and Hofmeister ions, hierarchically mesoporous MOFs (HMMOFs) with adjustable mesopore sizes are successfully achieved, which are competent to detect femtomolar level alkaline phosphatase (ALP). Such high sensitivity for ALP detection highlights the importance of adjustable mesopore structures of HMMOFs in advanced sensing applications, and prefigures their potential for biomedical diagnostics.

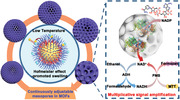

## 1 Introduction

Hierarchically mesoporous metal‐organic frameworks (HMMOFs) have attracted significant attention thanks to their exceptional properties and widespread applications.^[^ ^1^, ^2^, ^3^, ^4^, ^5^, ^6^, ^7^, ^8^, ^9^ ^]^ Driven by the demand for their capability to accommodate large biomolecules and to facilitate efficient mass transport, the facile construction of HMMOFs with precisely controlled large mesopores has become a highly desirable objective.^[^ ^10^, ^11^, ^12^, ^13^, ^14^, ^15^ ^]^ However, the achievement of such MOFs with continuously adjustable and uniform mesopore sizes ranging from 10 to 50 nm remains a significant challenge, prompting the need for the development of innovative synthesis methodology.

Microemulsion‐directed synthesis has emerged as a promising strategy for fabricating materials with diverse mesoporous architectures.^[^ ^16^, ^17^, ^18^, ^19^, ^20^, ^21^ ^]^ Microemulsion is usually formed by combining two immiscible liquid phases with surfactant micelles, where surfactant micelles envelop a hydrophobic solvent to create an oil‐in‐water system. Especially, many surfactant micelles featured temperature‐dependent swelling behavior. When the temperature is low enough, hydrophobic solvents can easily diffuse into the hydrophobic cores of the micelles, resulting in notable swelling and even alterations in their shapes.^[^ ^22^, ^23^ ^]^ Nevertheless, their swelling extent is usually uncontrollable with this low‐temperature avenue. Fortunately, Hofmerister salt ions were revealed to be competent to exert a significant influence on the phase behavior of block copolymers.^[^ ^24^, ^25^ ^]^ These salt ions typically bind to the hydrophilic layers of the micelles, affecting the diameter of the hydrophobic core and the thickness of the hydrophilic layer. Additionally, our previous works have verified that Hofmeister salt‐in ions, such as ClO_4_^−^, were able to enhance the interaction between MOF precursors and micelles, thereby facilitating the growth of MOF crystals in the outer poly(ethylene oxide) (EO) region of the micelles.^[^ ^21^, ^26^, ^27^ ^]^ This strongly inspires us to explore the synergistic effects between Hofmeister salt‐in ions and low‐temperature microemulsions, aiming to achieve continuous and tunable large mesopores in MOFs.

**Scheme 1:**
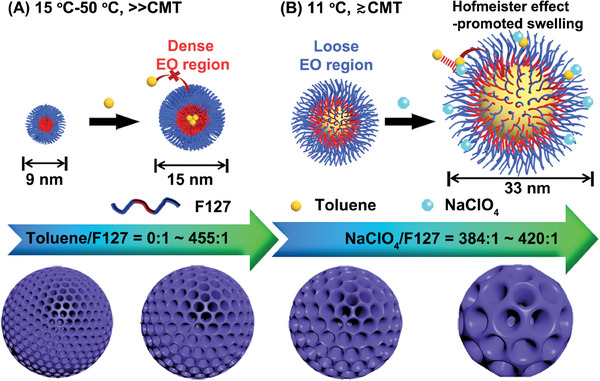
A schematic diagram illustrating the penetration of toluene into F127 micelles to form microemulsions at synthesis temperatures A) significantly above the critical microemulsion temperature (CMT) and B) slightly above the CMT. The size‐adjustable mesopores in MOFs are achieved by synergistically controlling the amounts of toluene and Hofmeister salt‐in ions of ClO_4_^−^.

Herein, we proposed a novel approach for the precise control of mesoporous sizes in HMMOFs promoted by Hofmeister salt‐in ions at low‐temperature, where F127 (EO_106_PO_70_EO_106_) triblock copolymer worked to form micelles and toluene was employed as a swelling agent. As expected, at traditional synthesis temperatures (15–50 °C), only a small amount of toluene can penetrate into the hydrophobic core of F127 micelles, resulting in a moderate mesopore expansion from 9 to 15 nm (**Scheme** **1**A). In contrast, at low temperature of 11 °C, much more toluene can penetrate, leading to a remarkable mesopore expansion of up to around 33 nm. Moreover, the synergistic effect of ClO_4_^−^ salt‐in ions enabled the continuous control of the mesopore expansion degree (Scheme 1B). By combining Hofmeister salt‐in effect and low temperature, we can precisely regulate the mesoporous sizes of HMMOFs within the range of 9–33 nm, offering great potential for tailoring their properties.

The large mesopores up to 33 nm enable the free diffusion of disease‐related protein markers (especially >10 nm), while ordered and accessible metal nodes in HMMOFs provide abundant sites for their efficient capture. Additionally, the spacious structure of large mesopores allows subsequent substrate diffusion, making HMMOFs competent to serve as an efficient biological reactor. Building upon these advantages, we successfully developed a colorimetric method for the femtomolar alkaline phosphatase (ALP) detection using the synthesized Ce‐based UiO‐66 type HMMOFs (HMUiO‐66(Ce)) as a versatile platform. The presence of large mesopores in HMUiO‐66(Ce) proved vital for the efficient enrichment of ALP and greatly enhanced sensitivity in ALP detection. By combining enrichment and cascade enzyme cycling amplification strategies, the developed sensing probe was able to detect low‐abundance ALP with a linear range of 100–7500 fm (equal to 0.000327–0.0245 U L^−1^) and limit of detection (LOD) of 42 fm (equal to 0.00014 U L^−1^), prefiguring their substantial potential for various applications, such as diagnostics and biomedical research.

## 2 Results and Discussion

### 2.1 HMUiO‐66(Ce) Synthesis and Characterization

**Figure 1:**
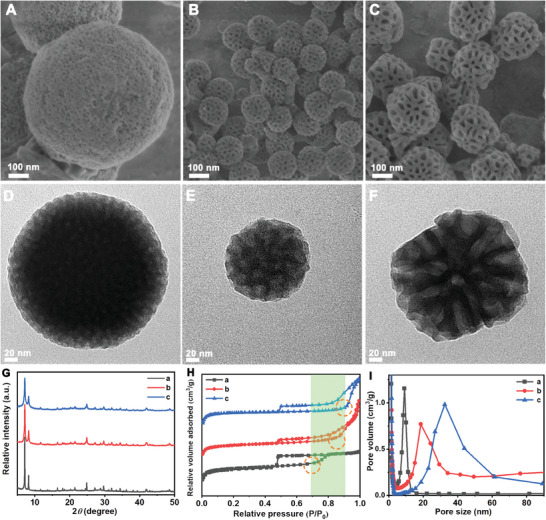
A–C) SEM and D–F) TEM images of HMUiO‐66(Ce) synthesized at 40 °C using F127 micelles A, D) and at 11 °C using toluene/F127 microemulsions as templates with NaClO_4_·H_2_O/F127 molar feed ratios of B, E) 315 and C, F) 420. G) XRD patterns, H) N_2_ sorption isotherms, and I) the corresponding pore size distribution curves of the representative HMUiO‐66(Ce) samples synthesized at 40 °C using F127 micelles (a, black line) and at 11 °C using toluene/F127 microemulsions as templates with NaClO_4_·H_2_O/F127 molar feed ratios of 315 (b, red line) and 420 (c, blue line), respectively.

In a typical synthesis, F127 was selected to form stable spherical micelles in the aqueous phase, enabling the growth of HMUiO‐66(Ce) through the mediation of Hofmeister ions.^[^ ^26^, ^27^ ^]^ Among these ions, ClO_4_^−^ was found as the optimal Hofmeister salt‐in ion for synthesizing HMUiO‐66(Ce) (Figure S1, Supporting Information). To adjust the mesopore size of HMUiO‐66(Ce) by enlarging the radius of F127 micelles, different amounts of hydrophobic toluene were introduced as a swelling agent. Subsequently, the synthesis of HMMOFs was guided by the swollen micelles at 40 °C. Scanning electron microscopy (SEM) images clearly demonstrated that the mesoporous entrance on the surface of HMUiO‐66(Ce) gradually expanded from ≈9 to 15 nm as the toluene/F127 molar feed ratios increased from 0 to 228 (**Figure** **1**A; Figure S2, Supporting Information). Further increasing the toluene/F127 feed ratios up to 455 would not lead to additional expansion of the mesopore. Interestingly, we discovered that low temperature was conducive to further expand mesopores. When the synthesis temperature was set at 11 °C, the HMUiO‐66(Ce) displayed a significant enlargement of the mesopore with a maximum size of ≈33 nm (Figure 1B,C). Transmission electron microscopy (TEM) images further revealed that all HMUiO‐66(Ce) samples possessed highly interconnected and size‐adjustable mesopores (Figure 1D–F; Figure S3, Supporting Information). It should be noted that the large microemulsion droplets at 11 °C become vulnerable to shear forces under stirring in the reaction, inducing a morphological shift from a spherical to an ellipsoidal shape. Therefore, the mesopores in HMUiO‐66(Ce)‐33 nm tend to be ellipsoidal shapes (Figure 1F). This mesoporous structure offers better connectivity, facilitating the diffusion of large biomarkers within the mesopores.

The X‐ray diffraction (XRD) analysis confirmed that all HMUiO‐66(Ce) samples exhibited well‐preserved diffraction peaks at 7.1^°^ and 8.2^°^ in the process of expanding mesopores (Figure 1G).^[^ ^28^ ^]^ This indicates that the inclusion of toluene at low‐temperature would not compromise the integrity of the crystalline walls. Then, the FT‐IR spectra of F127 and three HMUiO‐66(Ce) samples were investigated to demonstrate the complete removal of surfactants from HMUiO‐66(Ce) samples (Figure S4, Supporting Information). The FT‐IR signals at 2890 and 1116 cm^−1^ in the spectrum of F127 are attributed to the C─H stretching vibration and C─O─C stretching vibration of F127.^[^ ^29^ ^]^ After the adequate washing and activation, the disappearance of these peaks at 2890 and 1116 cm^−1^ in all of the HMUiO‐66(Ce) samples evidence the removal of surfactants after the activation treatment. The texture parameters of each sample were further characterized by N_2_ sorption isotherms, which displayed a combination of typical Type I and Type IV curves (Figure 1H; Table S1, Supporting Information). The adsorption branches displayed a rapid increase at low relative pressures (P/P_0_ = 0–0.05), indicating the presence of intrinsic and abundant micropores in the crystalline walls of HMUiO‐66(Ce). Moreover, the capillary condensation step progressively shifted to higher relative pressures (from 0.69 to 0.9), correlating with the significant expansion of mesopores. The mesopore size distribution was determined by the Barrett‐Joyner‐Halenda (BJH) model using the adsorption branch. At the synthetic temperature of 40 °C, the mesopore sizes of HMUiO‐66(Ce) could be only regulated from 9 to 15 nm upon increasing toluene/F127 molar feed ratios from 0 to 455 (Figure S5 and Table S2, Supporting Information). In contrast, at the low synthetic temperature of 11 °C, the mesopore size of HMUiO‐66(Ce) could be further modulated from 19 to 33 nm upon increasing NaClO_4_·H_2_O/F127 molar ratios from 315 to 420 (Figure 1I).

### 2.2 Mechanism of Mesopore Size Tunability

**Figure 2:**
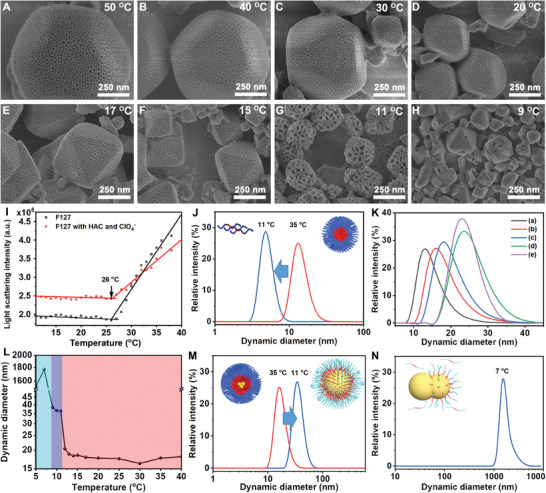
A–H) SEM images illustrates the relationship between the synthesis temperatures and the tunability of mesopores in HMUiO‐66(Ce). In the experiments, the molar feed ratios of toluene/F127 and NaClO_4_·H_2_O/F127 were kept constant at 455 and 420, respectively. I) Change in the light scattering intensity as a function of temperature for 16.7 mg mL^−1^ F127 solutions in the presence (red line) and absence of 582 mm NaClO_4_·H_2_O and 146 mm acetic acid (black line). J) The DLS profiles of F127 micelles in our synthesis system at 11 and 35 °C. K) The DLS profiles of F127 micelles at 35 °C with different toluene/F127 molar ratios of a) 0, b) 57, c) 114, d) 228, and e) 455, respectively. L) The dependence of the dynamic diameter of F127 microemulsion on temperatures. M, N) The DLS profiles of F127 microemulsion at 35 °C, 11 °C (M) and 7 °C (N).

To investigate the mechanism for constructing HMMOFs with very large mesopores, parallel experiments were conducted to disclose the impact of different synthesis temperatures in the presence of a constant amount of toluene and ClO_4_^−^. When the synthesis temperatures were set above 15 °C, only a slight enlargement in mesopore size was observed with increasing temperatures from 15 to 50 °C, as determined by SEM and BJH analysis (**Figure** **2**A–F; Figure S6 and Table S3, Supporting Information). This is reasonable since higher temperature generally leads to an increased aggregation number of F127 micelle, which rationalizes slight enlargement of the microemulsion size and corresponding replicated mesopores in HMMOFs.^[^ ^30^, ^31^ ^]^ In contrast, a significant increase in mesopore size up to 33 nm was visible when the synthesis temperature was decreased to 11 °C (Figure 2G). Nevertheless, when the synthesis temperature was further lowered to 9 °C, almost no mesopores were evolved (Figure 2H). Therefore, it can be concluded that the low‐temperature synthesis at 11 °C should be responsible for the successful introduction of large mesopores in HMMOFs. Furthermore, we conducted XRD measurements for HMUiO‐66(Ce) samples synthesized at various temperatures. It can be observed that HMUiO‐66(Ce) synthesized at 40 °C and 11 °C both exhibit a high degree of crystallinity (Figure S7, Supporting Information). Even after soaking these HMUiO‐66(Ce) samples in acidic or alkaline solutions for three days, they still maintained their intact crystallinity. Therefore, HMUiO‐66(Ce) synthesized using the low‐temperature synthesis strategy maintains both high crystallinity and excellent chemical stability.

To get insight into the decisive role of the low synthesis temperature played, we conducted a comprehensive investigation into the structural changes of F127 micelles at different temperatures. Light scattering experiments revealed that the critical micelle temperature (CMT) of F127 surfactant within our synthesis system was around 26 °C (Figure 2I).^[^ ^32^, ^33^ ^]^ Below this CMT, F127 copolymers mainly existed as 5 nm unimers,^[^ ^34^ ^]^ whereas above the CMT, F127 unimers underwent a transformation to spherical micelles with hydrodynamic diameter of around 14 nm (Figure 2J). When toluene was introduced into the system, it could penetrate the hydrophilic EO layer of the F127 micelles and mix with the hydrophobic poly(propylene oxide) (PO) core, thereby expanding the size of the original F127 micelles. Nevertheless, at a relatively high temperature (above 15 °C), the penetration of hydrophobic toluene into the micellar core was hindered due to the long hydrophilic block of F127 micelles (Scheme 1A).^[^ ^23^ ^]^ Consequently, only a limited amount of toluene was able to enter the PO core, resulting in a modest increase in the hydrodynamic diameter of F127 microemulsion from 14 to 24.8 nm (Figure 2K).

Then, we further investigated the temperature‐dependent transition process of F127 microemulsions using dynamic light scattering (DLS). At low temperatures, specifically when the toluene/F127 molar ratio exceeds 114, the concentration of the microemulsion becomes excessively high, making the solution become milky white. The light from the DLS device cannot penetrate the milky white microemulsion, which makes it difficult to test its hydrodynamic diameter. Consequently, we utilized the toluene/F127 molar ratio of 57 to investigate the dependence of the dynamic diameter of F127 microemulsion on temperature. In comparison to pure F127 micelles, the microemulsion exhibited a lower CMT of ≈9 °C attributed to the presence of hydrophobic toluene in the core of F127 microemulsion (Figure 2L), which enhanced its stability at low temperatures.^[^ ^35^ ^]^ A high correlation was observed between the temperature‐dependent behavior of F127 microemulsion sizes and the mesoporous changes of HMUiO‐66(Ce) as determined by SEM. Within the temperature range of 13 °C–40 °C, the hydrodynamic diameter of F127 microemulsion remained constant at ≈18 nm. In general, lowering the temperature reduces the aggregation numbers in micelles (resulting in a slight contraction of the microemulsion size) and simultaneously increases micellar hydration (leading to a slight expansion of external hydration layer). As a result, the net change in the hydrodynamic diameter of microemulsion was close to zero as determined by DLS.^[^ ^30^, ^31^ ^]^ However, when the temperature slightly surpassed the CMT of microemulsion (T = 11 °C), the hydrodynamic diameter of F127 microemulsion significantly expanded to ≈37 nm (Figure 2M). This expansion was ascribed to an accelerated dynamic exchange between the F127 layer on the microemulsion surface and the F127 monomers in the aqueous solution, resulting in a lower density of EO regions on the microemulsion surface. This less compact aggregation weakened the barrier of the hydrophilic EO part to the penetration of toluene, allowing a substantial amount of toluene to swell the microemulsion (Scheme 1).^[^ ^23^, ^36^, ^37^ ^]^ Finally, when the synthesis temperature was lower than the CMT of microemulsion (T<9 °C), the F127 layer on the surface of the microemulsion dissociated, leading to the formation of large toluene emulsion and F127 unimers (Figure 2N). This prevented the formation of mesopores in MOFs. Therefore, the low‐temperature synthesis strategy was closely associated with the CMT and enabled substantial enlargement of mesopores in MOFs.

**Figure 3:**
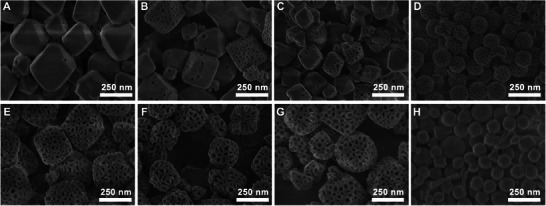
SEM images of HMUiO‐66(Ce) samples synthesized at a low temperature of 11 °C utilizing a toluene/F127 microemulsion as template. The structure of microemulsion was adjusted using various NaClO_4_·H_2_O/F127 molar feed ratios of A) 0, B) 105, C) 210, D) 315, E) 384, F) 420, G) 444, and H) 600, respectively.

Furthermore, Hofmeister salt‐in ions of ClO_4_^−^ are typically bound to the EO region of F127 microemulsion,^[^ ^24^, ^37^, ^38^ ^]^ thus influencing the structure of the microemulsion at 11 °C. In the absence of ClO_4_^−^, it could be observed that no mesopores are formed in the MOFs (**Figure** **3**A). Only a small amount of mesopores were generated when the NaClO_4_·H_2_O/F127 increased to 210 (Figure 3B,C). This phenomenon could be attributed to the crucial mediating effect of the Hofmeister salt‐in ions of ClO_4_^−^, which guided the growth of MOF crystals on the surface of microemulsion. Then, the mesopore sizes of HMUiO‐66(Ce) could be tuned between 19 and 33 nm by adjusting the NaClO_4_·H_2_O/F127 molar feed ratios within the range of 315 to 420 (Figure 3D–F). Due to the Hofmeister effect of ClO_4_^−^ salt‐in ions, it could improve the solubility of hydrophobic molecules in the hydrophilic media.^[^ ^39^, ^40^ ^]^ Consequently, the addition of appropriate NaClO_4_·H_2_O could further modulate the permeability of toluene across the EO region and effectively control the size of the microemulsion at low temperature. However, when the NaClO_4_·H_2_O/F127 was further increased from 444 to 600, the mesopores in the MOFs no longer expanded and eventually disappeared (Figure 3G,H). The excessive introduction of ClO_4_^−^ made the hydrophilic layer of F127 microemulsion excessively permeable, which in turn led to the instability and dissociation of the microemulsion. As a comparative experiment, we also studied the changes in mesoporous structure of HMUiO‐66(Ce) synthesized at 11 °C utilizing various toluene/F127 molar ratios (Figure S8, Supporting Information). As the molar ratio of toluene/F127 increases from 0 to 455, it could be observed that the number of formed mesopores in the HMUiO‐66(Ce) gradually increases. However, increasing the amount of toluene would not have a significant influence on the mesoporous size in HMUiO‐66(Ce). This means that adjusting the molar ratio of toluene/F127 alone only affects the number of microemulsion and cannot effectively regulate the size of the microemulsion at 11 °C. Therefore, the size‐adjustable mesopores in HMUiO‐66(Ce) could be achieved by the synergistic effects between Hofmeister salt‐in ions of ClO_4_^−^ and low‐temperature toluene/F127 microemulsions (Scheme 1), allowing for the screening of MOFs with customized mesopore sizes to meet the specific requirements of different dimensional biological macromolecules.

### 2.3 Enrichment and Cycling Amplification Strategies for Femtomolar ALP Detection

ALP is a natural enzyme with the capability to catalyze the dephosphorylation of phosphate esters. It plays a crucial role in a wide range of biological processes, including various metabolic pathways, signal transduction, and cellular regulation.^[^ ^41^ ^]^ Given its association with numerous diseases,^[^ ^42^ ^]^ abnormalities in ALP activity serve as important biomarkers for clinical diagnosis and treatment.^[^ ^43^, ^44^, ^45^, ^46^, ^47^, ^48^, ^49^, ^50^ ^]^ Hence, the visual detection of low abundance biomarker of ALP has garnered significant attention in the fields of diagnostics and biomedical research.

**Scheme 2:**
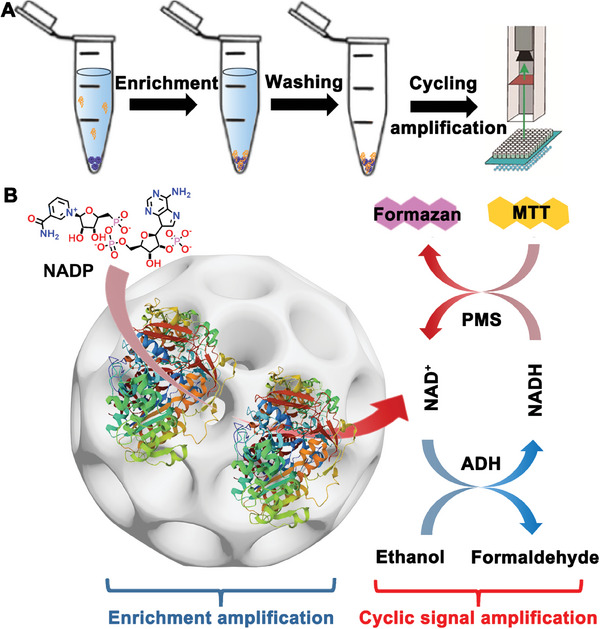
The schematic diagram illustrates A) the capture and enrichment of ALP using HMUiO‐66(Ce) and B) the subsequent multiplication‐level colorimetric signal amplification through a two‐step enzyme cycling reaction.

Using HMUiO‐66(Ce) with various mesopore sizes as a versatile platform, we have successfully realized the colorimetric detection of femtomolar levels of ALP by combining enrichment and cascade enzyme cycling amplification strategies. First, the presence of large mesopores in HMUiO‐66(Ce) is crucial for facilitating the diffusion of disease‐related protein markers, especially those larger than 10 nm. Furthermore, Ce‐based MOFs possess abundant Ce─OH sites, which have a strong affinity for the combination with phosphate and carboxylic groups in protein.^[^ ^26^, ^51^ ^]^ Thus, the ordered arranged metal nodes within HMUiO‐66(Ce) provide abundant and accessible binding sites for ALP, enabling the efficient capture and enrichment of trace amounts of ALP biomarkers (**Scheme** **2**A). Due to the electric neutrality of HMUiO‐66(Ce) under neutral pH conditions (Figure S9, Supporting Information), electrostatic interaction between HMUiO‐66(Ce) and ALP is very weak. Furthermore, the open mesoporous structure of HMUiO‐66(Ce) allows for effective substrate diffusion and exchange, ensuring optimal conditions for subsequent enzyme‐catalyzed colorimetric reactions. To magnify the detectable signal, we employ a two‐step cascade enzyme cycling reaction. In the initial enzymatic step, each enriched ALP catalyzes the dephosphorylation of nicotinamide adenine dinucleotide phosphate (NADP), leading to the generation of nicotinamide adenine dinucleotide (NAD) cofactors (Scheme 2B). The NAD cofactors play a vital role in the subsequent NAD‐dependent enzyme cycling reaction. In the cycling reaction, ethanol dehydrogenase (ADH) converts NAD cofactors to NADH, which is then regenerated to NAD through the electron acceptor of phenazine methosulfate (PMS). The electron‐accepting PMS intermediate rapidly oxidizes yellow 3‐(4,5‐dimethyl‐2‐thiazolyl)−2,5‐diphenyl‐2‐H‐tetrazolium bromide (MTT), resulting in the production of purple formazan, which can be quantitatively measured by the absorbance at 570 nm. In this continuous cycling process, each NAD can produce a large number of formazan dyes, resulting in a multiplicative amplification effect that significantly enhances the detectable signal. As a result, this innovative method offers improved sensitivity for detecting ultralow levels of ALP. Obviously, the large mesopores up to 33 nm not only guarantee the enrichment of ALP biomarkers but also provide sufficient space for the ALP‐catalytic colorimetric response process.

**Figure 4:**
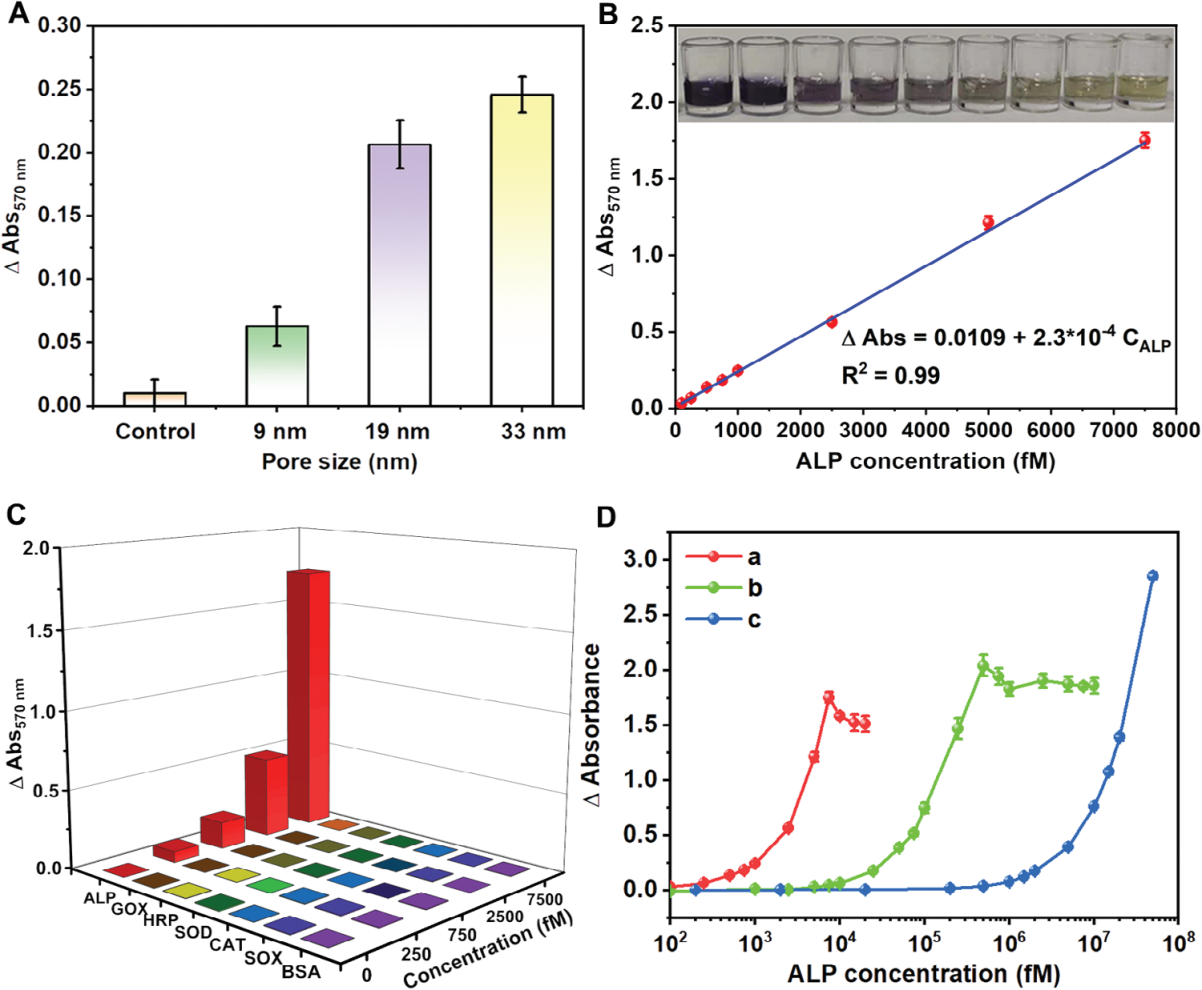
A) Sensing response of HMUiO‐66(Ce) with different mesopore sizes based on the absorbance (λ = 570 nm) variations from the product of formazan toward 1000 fm ALP using the proposed cascade enzyme cycling amplification strategy. B) The change of absorbance plotted against ALP concentrations, along with the corresponding linear fitting curve. The inset shows the visible color change from yellow to purple as ALP concentration stepwise increased from 0, 100, 250, 500, 750, 1000, 2500, 5000, to 7500 fm. C) Sensing response of HMUiO‐66(Ce) against various possibly coexistent interfering species (0–7500 fm) based on the cycling amplification strategy. Glucose oxidase, horseradish peroxidase, superoxide dismutase, catalase, sulfite oxidase and bovine serum albumin were abbreviated as GOX, HRP, SOD, CAT, SOX and BSA, respectively. D) Comparison of the response properties of cyclic amplification methods in the presence (red line) and absence of HMUiO‐66(Ce) (green line), and classic PNPP colorimetric method (blue line).

To validate our proposal, we first compared the response of HMUiO‐66(Ce) with different mesopore sizes toward ALP. As shown in **Figure** **4**A, the absence of HMUiO‐66(Ce) leads to negligible enhancement in absorbance at 570 nm from formazan, while such absorbance is greatly enhanced upon the introduction of HMUiO‐66(Ce). Moreover, their absorbance response amplitudes are positively correlated to the mesopore sizes of HMUiO‐66(Ce), making larger mesopores more effective in detecting low‐abundance ALP. Given that the 3D parameters of ALP are 10.0 nm × 5.0 nm × 5.0 nm,^[^ ^52^ ^]^ it becomes apparent that HMMOFs with larger mesopores are necessary to provide sufficient space for ALP to freely diffuse and anchor within the interior of HMMOFs. Thus, we further utilized HMUiO‐66(Ce) with a mesoporous size of 33 nm to investigate their sensory properties toward ALP detection.

Based on the enrichment and cyclic amplification with HMUiO‐66(Ce), a significant enhancement in absorbance at 570 nm, which can be attributed to the formation of formazan, was observed as the ALP concentration increased within the range of 100 fm‐7500 fm (equal to 0.000327–0.0245 U L^−1^). A linear curve could be fitted by plotting the enhanced absorbance values against the corresponding ALP concentrations (Figure 4B). By employing the 3σ rule (Figure S10, Supporting Information), the LOD was determined to be ≈42 fm (equal to 0.00014 U L^−1^). Additionally, the digital photos in Figure 4B displayed the color transition of the solution from yellow to purple as the ALP concentration increased, in line with the observed absorbance change. Meanwhile, the color transition enabled the naked eye to detect ALP concentrations as low as around 500 fm. Subsequently, we performed comprehensive tests to evaluate the influence of various potential interfering species on the sensing system, including GOX, HRP, SOD, CAT, SOX, and BSA. Negligible responses were observed for all these possibly coexistent interfering biomolecules, while an obvious absorbance enhancement at 570 nm could be visible upon the introduction of ALP into the sensory system (Figure 4C). Meanwhile, no obvious impacts on the sensing response occurred when these interfering species were simultaneously added to the ALP‐containing detecting systems (Figure S11, Supporting Information).

To testify the advantages of this approach, we also compared the developed sensory strategy to the single cyclic amplification method without HMMOFs, and classic para‐nitrophenyl phosphate (PNPP) colorimetric method. PNPP is a widely used phosphatase chromogenic substrate in commercial assay kits. Under alkaline conditions, it can be converted into p‐nitrophenol by ALP, and the absorbance of p‐nitrophenol can be measured at 405 nm. It could be observed that the classic colorimetric assay kit was capable of detecting ALP within the linear range of 2 × 10^5^–5 × 10^7^ fm (Figure 4D, blue line; Figure S12, Supporting Information). Meanwhile, the single cyclic amplification method without HMMOFs could detect ALP within the linear range of 5 × 10^3^–2.5 × 10^5^ fm (Figure 4D, green line; Figure S13, Supporting Information). In comparison, our developed method based on the enrichment and cyclic amplification with HMUiO‐66(Ce) exhibited a significant enhancement in absorbance at 570 nm with increasing ALP concentration in the range of 100–7500 fm, reaching a plateau beyond 7500 fm (Figure 4D, red line). There is a significant improvement in the LOD (Figures S14 and S15, Supporting Information) for the currently developed sensory probe, with ≈61 and 4423‐fold improvement compared to the single cyclic amplification (LOD = 2571 fm), and the classic PNPP colorimetric methods (LOD = 185777 fm), respectively. In addition, in comparison to the recently reported colorimetric methods for ALP detection, the proposed approach also exhibits higher sensitivity, even surpassing most reported fluorescence‐based detection methods (Table S4, Supporting Information). Such high sensitivity for low‐abundance ALP detection prefigures that the developed HMMOFs‐based sensory platform might hold broad application prospects in fields such as disease diagnosis and biomedical research.

Finally, to validate our method's capacity to detect ALP concentration in practical clinical samples, we collected serum samples from four volunteers for the comparison of detection of ALP by using the commercialized ALP detection kit and our developed method. First, we utilized a commercial ALP test kit for the detection of ALP, in which serum samples were diluted tenfold with a testing buffer to meet the ALP detection range of the commercial kit. The ALP activity in the human serum samples was measured to be between 99.4–117.1 U L^−1^ by the commercial ALP detection kit (Figure S16 and Table S5, Supporting Information). Subsequently, we used our developed method for the ALP test in serum samples, which were diluted 10^5^‐fold with a buffer solution to ensure that the ALP activity concentration aligned with our method's linear detection range (Figure S17, Supporting Information). The measured serum ALP activity concentrations were close to the value obtained using the commercial alkaline phosphatase test kit (Table S5, Supporting Information). Furthermore, when extra ALP (100, 300, and 600 U L^−1^) was added to the serum samples, the detected ALP activity concentrations also matched well with the added ALP amounts, with a recovery rate fluctuating between ≈92.8% and 102.1% (Table S6, Supporting Information). Therefore, the developed ALP detection method demonstrates the effectiveness of detecting ALP in actual serum samples, while requiring only a very small amount of serum sample (0.5 µL) to satisfy the testing requirements thanks to its high sensitivity.

## 3 Conclusion

In summary, by combining the synergistic effects of low‐temperature microemulsions and Hofmeister salt‐in ions, HMMOFs with continuously tunable mesoporous sizes ranging from 9 to 33 nm were successfully achieved. The low‐temperature just above the CMT of F127 microemulsion significantly enhanced the permeability of toluene into F127 micelles, resulting in substantial swelling of microemulsion and the formation of large mesopores within the HMUiO‐66(Ce). Moreover, the Hofmeister effect of ClO_4_^−^ salt‐in ions could regulate the permeability of toluene into the microemulsion, thereby continuously controlling the swelling degree of microemulsion. The large mesopores up to 33 nm not only facilitated the free diffusion of disease‐related ALP biomarkers in HMUiO‐66(Ce), but also provided abundant Ce─OH sites for their efficient capture. Furthermore, the remaining space in the mesopores still satisfied the substrate diffusion in subsequent colorimetric reactions. On the basis of these advantages, the resulting HMUiO‐66(Ce) could detect femtomolar ALP through enrichment and cycling amplification strategies, with a linear detection range of 100–7500 fm and a LOD of 42 fm. The LOD of HMUiO‐66(Ce) sensor presented around 61 and 4423‐fold improvement compared to the single cyclic amplification and the classic PNPP colorimetric methods, respectively. Such sensitivity in detecting femtomolar ALP indicates the substantial potential of this sensory platform in disease diagnostics and biomedical research aspects. Furthermore, the ability to finely tune mesoporous sizes might pave the way for designing HMMOFs with tailored properties and functionalities to meet diverse application needs.

## 4 Experimental Section

### The Modulation of Mesoporous Sizes of HMUiO‐66(Ce) at Normal Temperatures

F127 (100 mg, 0.00833 mmol) was dissolved in 6 mL of deionized water. Subsequently, acetic acid (HAc) (0.05 mL, 0.85 mmol), different amounts of toluene (toluene/F127 molar feed ratios of 0, 57, 114, 228, and 455), and NaClO_4_·H_2_O (490 mg, 3.5 mmol) were added. The mixtures were stirred for 1 h to obtain a homogeneous solution. Then, 1,4‐dicarboxybenzene (BDC) (166 mg, 1 mmol) and (NH_4_)_2_Ce(NO_3_)_6_ (548 mg, 1 mmol) were added to the solution. The reaction mixtures were stirred at 15 °C to 50 °C in a water bath for 40 min. Afterward, the resulting samples were centrifuged and washed twice with water, followed by two washes with DMSO to remove the residual unreacted ligands. Then, the synthesized samples were washed with ethanol and immersed in ethanol at 60 °C for two days, with daily replacement of the ethanol. Finally, the samples were subjected to vacuum drying at 80 °C for 24 h.

### The Modulation of Large Mesoporous Sizes of HMUiO‐66(Ce) at Low‐Temperature

F127 (100 mg, 0.00833 mmol) was dissolved in 6 mL of deionized water. Subsequently, HAc (0.05 mL, 0.85 mmol), toluene (400 µL), and different amounts of NaClO_4_·H_2_O (NaClO_4_·H_2_O/F127 molar ratios of 0, 105, 210, 315, 384, 420, 444, and 600) were added to the solution. The resulting mixture was subjected to stirring under a constant temperature water bath set at 11 °C for 18 h, forming a homogeneous microemulsion. Then, BDC (166 mg, 1 mmol) and (NH_4_)_2_Ce(NO_3_)_6_ (548 mg, 1 mmol) were added to the solution, followed by stirring at 11 °C for 4 h. Subsequently, the obtained samples were centrifuged and washed twice with water. This was followed by two additional washes with DMSO to remove any remaining unreacted ligands. The synthesized samples were then washed with ethanol and immersed in ethanol at 60 °C for two days, with daily replacement of the ethanol. Finally, the samples underwent vacuum drying at 80 °C for 24 h.

### Light Scattering Studies for the CMT

The CMT of F127 samples was determined with static light scattering using a RF‐6000 spectro‐fluorophotometer at an excitation wavelength of 520 nm. F127 solution (100 mg in 6 mL deionized water) and F127 solutions with similar conditions for MOFs synthesis (100 mg in 6 mL deionized water, containing 0.05 mL HAc and 490 mg NaClO_4_·H_2_O) were subjected to stirring as temperatures gradually increased from 11 °C to 40 °C. As the temperature increased, the intensity of the Rayleigh band (520 nm) of F127 samples was measured as a function of temperature. The temperature at which the scattering intensity abruptly increased was determined as the CMT.

### Colorimetric Detection of ALP in the Presence or Absence of HMMOFs

Preparation of HMMOFs‐immobilized assay tubes: 10 mL of BSA blocking solution (0.5 wt.%, in PBS buffer, pH 7.4) was added to each 10 mL centrifuge tube, and incubated at 37 °C for 2 h. The tubes were then washed three times with Tris‐HCl buffer (20 mm, pH 7.4), followed by removing residual liquid. Subsequently, 50 µL of HMUiO‐66(Ce) suspension (25 mg L^−1^, in ethanol) was added to the bottom of each tube. The assay tubes were placed in a vacuum drying oven at 37 °C for 24 h to obtain the MOFs‐immobilized assay tubes.

ALP detection: 10 mL of ALP samples with various concentrations were added to the MOFs‐immobilized assay tubes. The samples were allowed to incubate for 1 h to enrich ALP, followed by washing three times with Tris‐HCl buffer (20 mm, pH 7.4). Then, 150 µL of 0.2 mm NADP in diethanolamine buffer (50 mm diethanolamine, pH 9.5, containing 1 mm Mg^2+^ and 1 µm Zn^2+^) was added to each assay tube, and incubated at 37 °C for 3 h. Subsequently, 50 µL of the liquid from each tube was transferred to a 96‐well plate, and then 50 µL of the cyclic amplification colorimetric solution was added to each well. The plate was incubated at 37 °C for 10 min, and the absorbance at 570 nm was measured using a microplate reader. The cyclic amplification colorimetric solution was prepared by adding PMS, MTT, ADH, and ethanol to Tris‐HCl buffer. The mixture was used immediately after thorough mixing. For example, when testing 48 samples, 490 µL of PMS (4.2 mm), 490 µL of MTT (10.5 mm), 490 µL of ADH (4.2 mg mL^−1^), and 389 µL of ethanol were added to 3312 µL of Tris‐HCl buffer (100 mm, pH 8) in order to prepare the required amount of the cyclic amplification colorimetric solution.

The single cyclic amplification colorimetric ALP detection was carried out with the same procedure in the absence of HMMOFs.

### Colorimetric Detection of ALP Based on Classical PNPP Method

10 uL of ALP solution was added to 96‐well plate, and then 90 µL of 1.11 mm PNPP in DEA buffer (50 mm diethanolamine, pH 9.8, containing 1 mm Mg^2+^) was added to each well. The ALP concentration refers to the final concentration after mixing in the well plate. Then, the plate was incubated at 37 °C for 10 min, and the absorbance at 405 nm was measured using a microplate reader.

## Conflict of Interest

The authors declare no conflict of interest.

## Supporting information

Supporting Information

## Data Availability

The data that support the findings of this study are available from the corresponding author upon reasonable request.
