# Supplementary material for: Hofmeister Effect Promoted the Introduction of Tunable Large Mesopores in MOFs at Low Temperature for Femtomolar ALP Detection
Source: Adv Sci (Weinh). 2023 Nov 30;11(4):2305786. doi: 10.1002/advs.202305786 (PMC10811466; doi:10.1002/advs.202305786)
Supplement: Supplementary file 1 — Supporting Information [file ADVS-11-2305786-s001.pdf]

ADVANCED SCIENCE
Open Access

Supporting Information

for *Adv. Sci.*, DOI 10.1002/advs.202305786

Hofmeister Effect Promoted the Introduction of Tunable Large Mesopores in MOFs at Low Temperature for Femtomolar ALP Detection

*Jian Yang, Ming Gong, Fan Xia, Yao Tong and Jinlou Gu**

Supporting Information

Hofmeister Effect Promoted the Introduction of Tunable Large Mesopores in MOFs at Low Temperature for Femtomolar ALP Detection

*Jian Yang, Ming Gong, Fan Xia, Yao Tong and Jinlou Gu**

Key Laboratory for Ultrafine Materials of Ministry of Education, School of Materials Science and Engineering, East China University of Science and Technology, Shanghai 200237, China

*E-mail: **jinlougu@ecust.edu.cn.** Fax: +86-21-64250740. Tel: +86-21-64252599 **(J. L. Gu)**

S1. Chemicals and Materials

Cerium(IV) ammonium nitrate ($(NH_4)_2Ce(NO_3)_6$), tris(hydroxymethyl)aminomethane (Tris), diethanolamine, nicotinamide adenine dinucleotide phosphate (NADP) and 3-(4,5-dimethyl-2-thiazolyl)-2,5-diphenyl-2-H-tetrazolium bromide (MTT) were purchased from Shanghai Macklin Biochemical Co., Ltd. Sodium perchlorate monohydrate ($NaClO_4 \cdot H_2O$), toluene, DMSO, ethanol, acetic acid (HAC), $MgCl_2 \cdot 6H_2O$ and $ZnCl_2$ were purchased from Sinopharm Chemical Reagent Co., Ltd. F127 and alcohol dehydrogenase (ADH) was supplied by Sigma-Aldrich. 1,4-dicarboxybenzene (BDC) was purchased from Shanghai Aladdin Biochemical Technology Co., Ltd. bovine serum albumin (BSA) (IgG, Protease, DNAse Free) was purchased from Yeasen Biotech Co., Ltd. Alkaline phosphatase (ALP, 38 U/mg) and phenazine methosulfate (PMS) were purchased from Bide Pharmatech Co., Ltd. sulfite oxidase (SOX) was purchased from J&K Scientific Ltd., China. Para-nitrophenyl phosphate (PNPP), glucose oxidase (GOX), horseradish peroxidase (HRP), superoxide dismutase(SOD), catalase (CAT) were purchased from Shanghai yuanye Bio-Technology Co., Ltd. The commercial ALP test kit was purchased from Shanghai Biyuntian Biological Co., Ltd. The applied water (18.1 $M\Omega \cdot cm^{-1}$) in the experiments was purified from a NW Ultrapure Water System (Heal Force, China).

S2. Instruments and Methods

The powder X-ray diffraction (XRD) patterns were obtained on a Bruker D8 instrument using Cu Kα radiation (40 kV, 40 mA). N_2 sorption isotherm was recorded using a surface area and pore size analyzer (Micromeritics Tristar 3020). The sample was degassed under vacuum at 100 ^oC for 12 h prior to analysis. The specific surface area was calculated by the Brunauer-Emmett-Teller (BET) method using adsorption data. The field emission scanning electron microscopy (SEM) was conducted on Hitachi S-4800. Transmission electron microscopy (TEM) was conducted on a JEM-1400 electron microscope. Light Scattering

Studies were recorded with an RF-6000 spectro-fluorophotometer (Shimadzu). The dynamic light scattering (DLS) was measured with a ZEN3700 system. For the detection of ALP based on the colorimetric array, Thermo scientific microplate reader was used to determine the absorption value of each sample.

S3. Results and Discussion

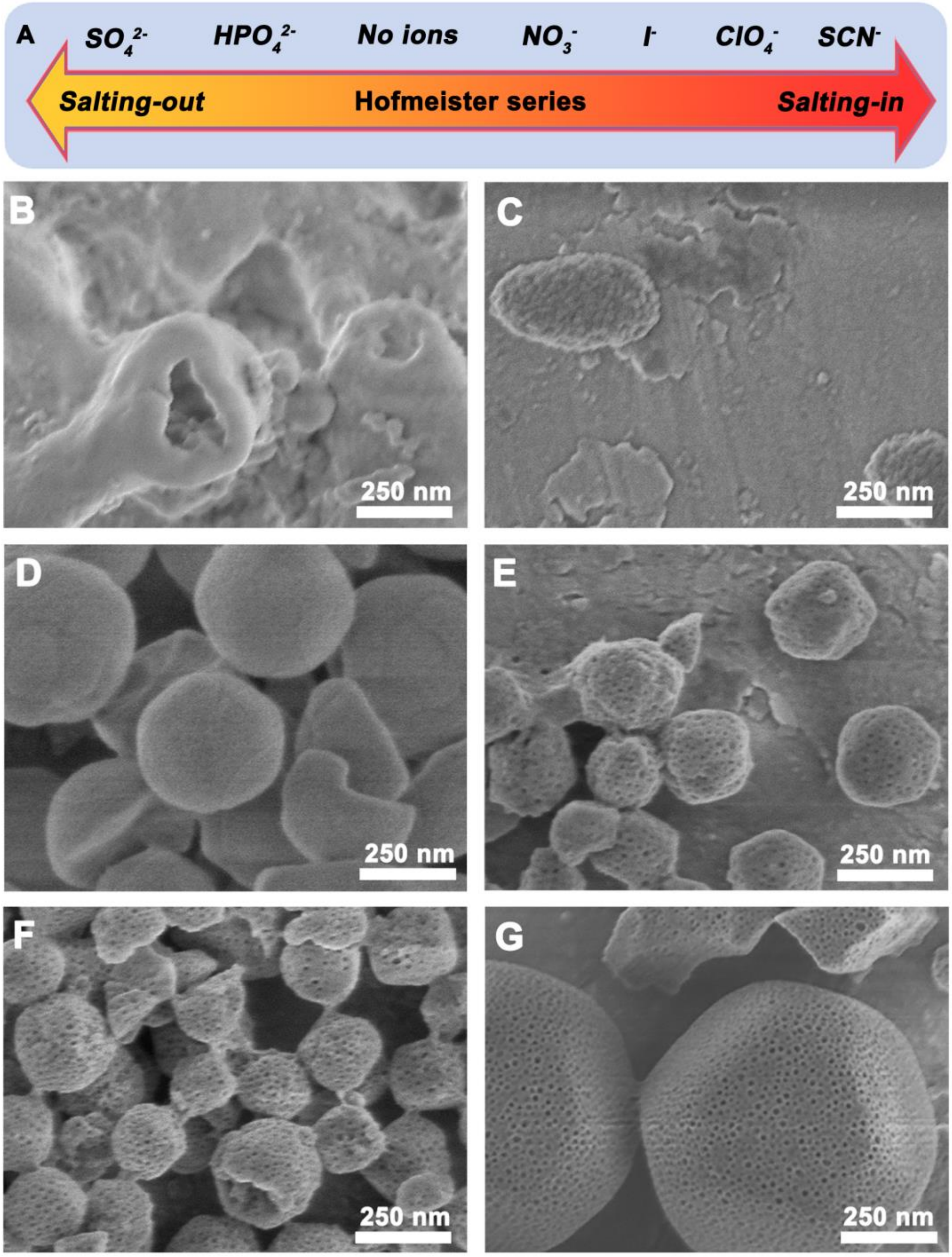

Figure S1. (A) Schematic diagram of traditional Hofmeister series. (B-G) SEM images of

HMUiO-66(Ce) samples synthesized at 40 ^{o}C with various Hofmeister salt ions utilizing toluene/F127 microemulsions as templates. The toluene/F127 molar ratio was set as 455, and the Hofmeister salt ions were (B) SO_4^{2-}, (C) HPO_4^{2-}, (D) no salt ions, (E) NO_3^-, (F) I^- and (G) ClO_4^-. It could be observed that no mesopores were formed in the MOFs when salting-out ions such as SO_4^{2-} and HPO_4^{2-} were employed (**Figures S1B and C**). When no salt-in ions were added, almost no mesopores appeared on the surface of HMUiO-66(Ce) (**Figure S1D**). Upon introducing salt-in ions, as the salt-in capability increased (in the order $NO_3^- < I^- < ClO_4^-$), the quantity and orderliness of the mesopores in the HMUiO-66(Ce) gradually improved (**Figures S1E-G**). This can be attributed to that the salt-in ions enhanced the interaction between the MOFs and micelles, thereby promoting the growth of MOF crystals on the micelle surface and leading to the formation of an ordered mesoporous structure. On the other hand, while NaSCN possess higher salt-in capabilities, it would decompose under acidic conditions to produce the highly toxic HCN gas. Therefore, $NaClO_4$ was the preferred choice as the salt-in ion for the synthesis of mesoporous MOFs.

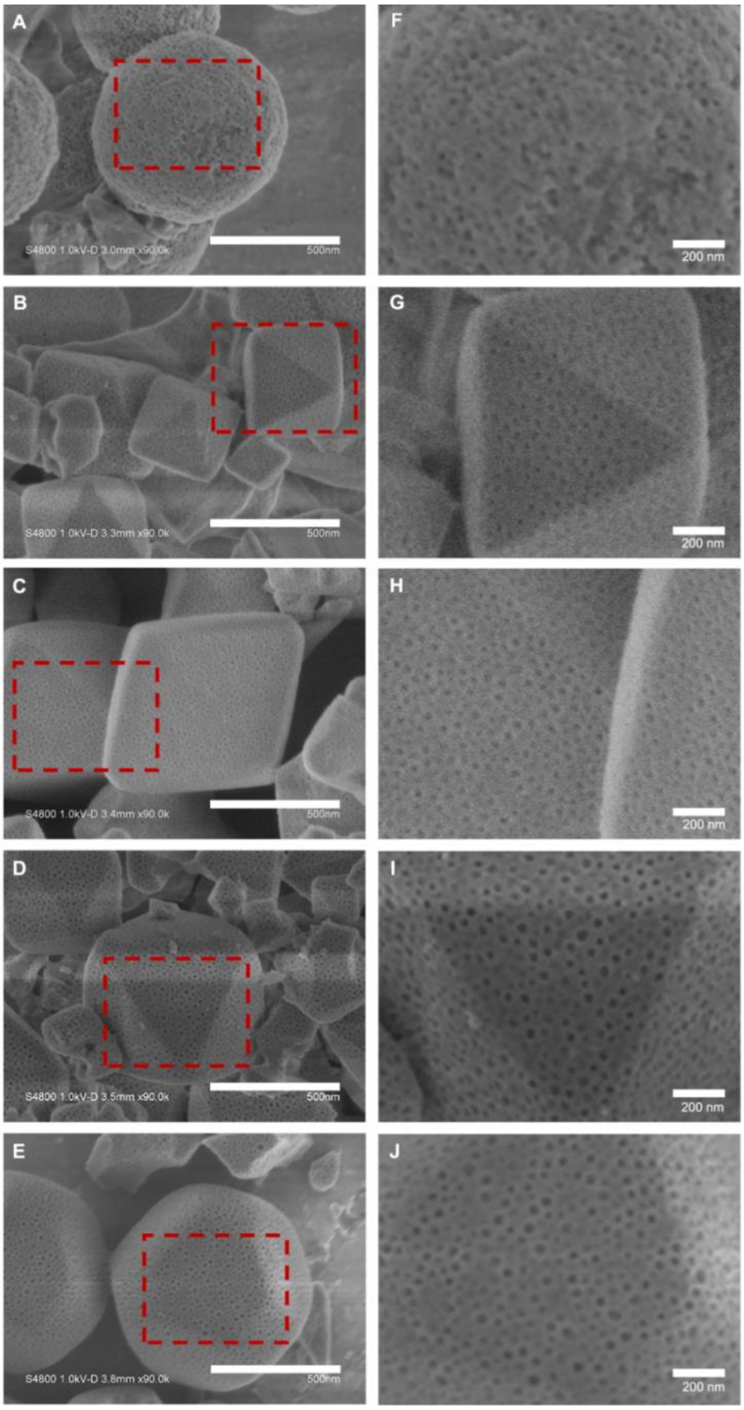

Figure S2. (A-E) SEM and (F-J) their corresponding enlarged images in the red selection area for HMUiO-66(Ce) samples synthesized at 40 ^{o}C utilizing toluene/F127 microemulsions as templates. The toluene/F127 molar ratios were set as (A, F) 0, (B, G) 57, (C, H) 114, (D, I) 228, and (E, J) 455, respectively.

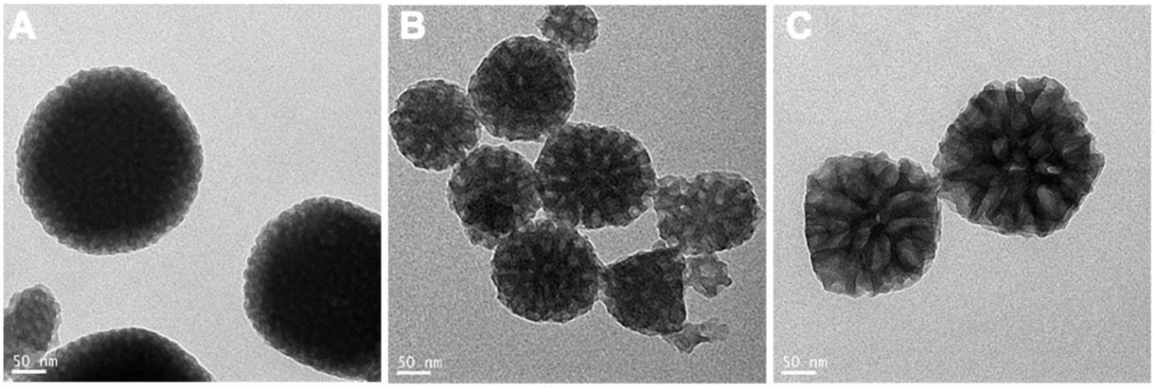

Figure S3. (A-C) TEM images of HMUiO-66(Ce) at a larger scale, providing visual evidence of the presence of interconnected mesopores within each particle. (A) In the first method, the synthesis was carried out at 40 °C using F127 micelles. In the second method, HMUiO-66(Ce) samples were synthesized at 11 °C using toluene/F127 microemulsions with adjusted $NaClO_4 \cdot H_2O$/F127 feed ratios of (B) 315 and (C) 420, respectively.

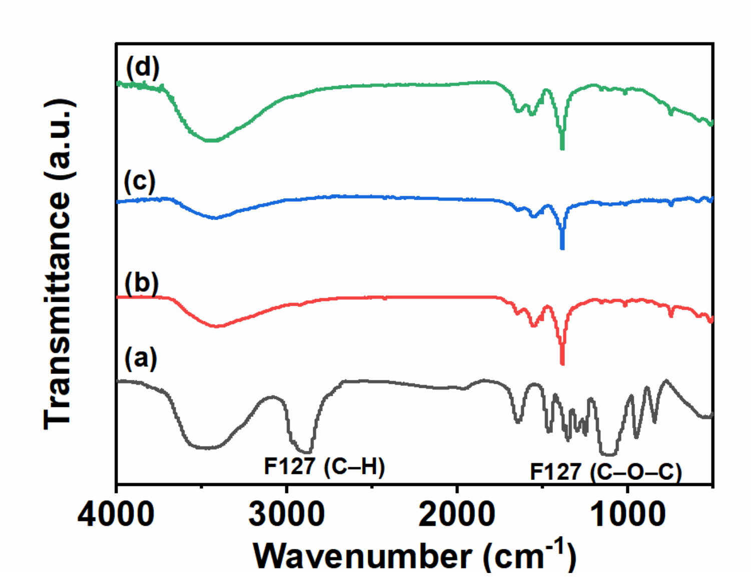

Figure S4. FT-IR spectra of (a) F127, (b) HMUiO-66(Ce)-9nm, (c) HMUiO-66(Ce)-19nm and (d) HMUiO-66(Ce)-33nm samples.

Table S1. Textural parameters for the representative HMUiO-66(Ce) samples synthesized at different temperatures.

Samples	D_{meso} (nm)	S_{BET} (m^2/g)	V_P (cm^3/g)
**HMUiO-66(Ce)-9nm**[a]	9	1194	0.64
**HMUiO-66(Ce)-19nm**[b]	19	904	0.85
**HMUiO-66(Ce)-33nm**[c]	33	1090	0.80

[a]*The HMUiO-66(Ce)-9nm sample was synthesized at 40 °C using F127 micelles.*

[b,c]*The HMUiO-66(Ce)-19nm and HMUiO-66(Ce)-33nm were synthesized at 11 °C using toluene/F127 microemulsions with adjusted $NaClO_4{\cdot}H_2O$/F127 molar feed ratios of 315 and 420, respectively.*

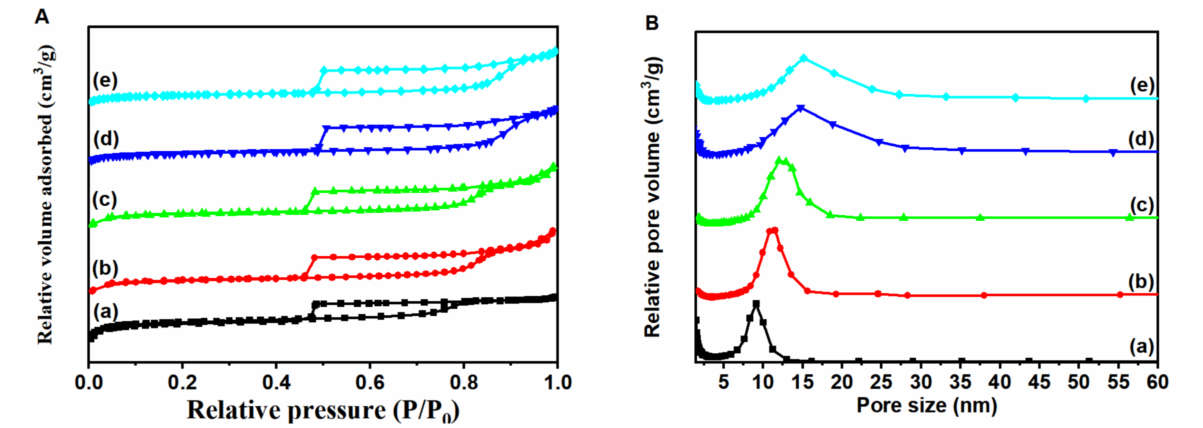

Figure S5. (A) N_2 sorption isotherms and (B) their corresponding pore size distribution curves for HMUiO-66(Ce) samples synthesized at 40 °C utilizing toluene/F127 microemulsions as templates. The microemulsions were adjusted using various toluene/F127 molar feed ratios of (a, black line) 0, (b, red line) 57, (c green line) 114, (d, blue line) 228, and (e, cyan line) 455, respectively.

Table S2. Textural parameters for the HMUiO-66(Ce) samples synthesized at 40 °C, utilizing toluene/F127 microemulsions. The microemulsions were adjusted using various toluene/F127 molar feed ratios.

Toluene/F127	D_{meso} (nm)	S_{BET} (m^2/g)	V_P (cm^3/g)
**0**	9	1194	0.64
**57**	11	935	0.71
**114**	13	880	0.70
**228**	15	726	0.67
**455**	15	632	0.58

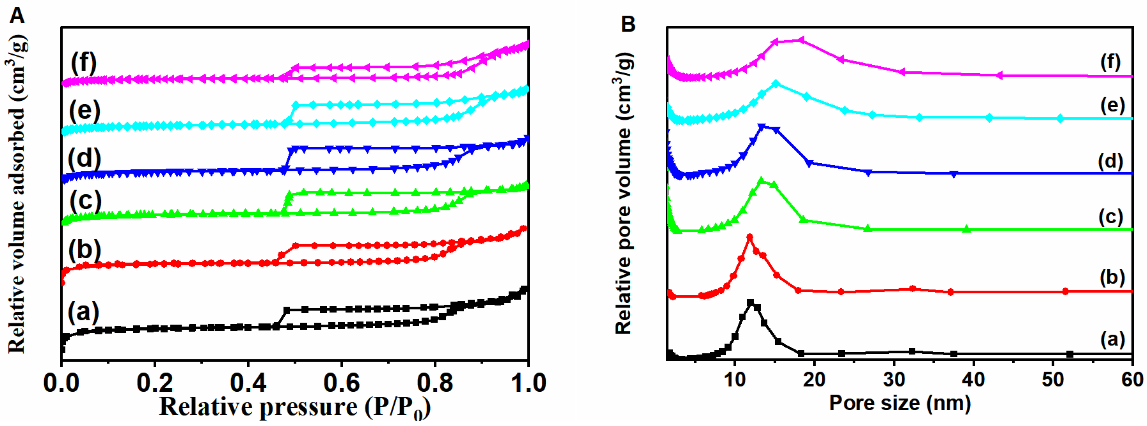

Figure S6. (A) N_2 sorption isotherms and (B) their corresponding pore size distribution curves for HMUiO-66(Ce) samples synthesized at different temperatures of (a, black line) 15 °C, (b, red line) 17 °C, (c green line) 20 °C, (d, blue line) 30 °C, (e, cyan line) 40 °C, and (f, purple) 50 °C using toluene/F127 microemulsions. In the experiment, the molar feed ratios of toluene/F127 and $NaClO_4 \cdot H_2O$/F127 were kept constant at 455 and 420, respectively.

Table S3. Textural parameters for the HMUiO-66(Ce) samples synthesized at different temperatures using toluene/F127 microemulsions. In the experiment, the molar feed ratios of toluene/F127 and $NaClO_4 \cdot H_2O$/F127 were kept constant at 455 and 420, respectively.

Synthesis temperature (°C)	D_{meso} (nm)	S_{BET} (m^2/g)	V_P (cm^3/g)
**15**	12	865	0.64
**17**	12	935	0.71
**20**	13	823	0.62
**30**	13	766	0.59
**40**	15	632	0.58
**50**	16	521	0.52

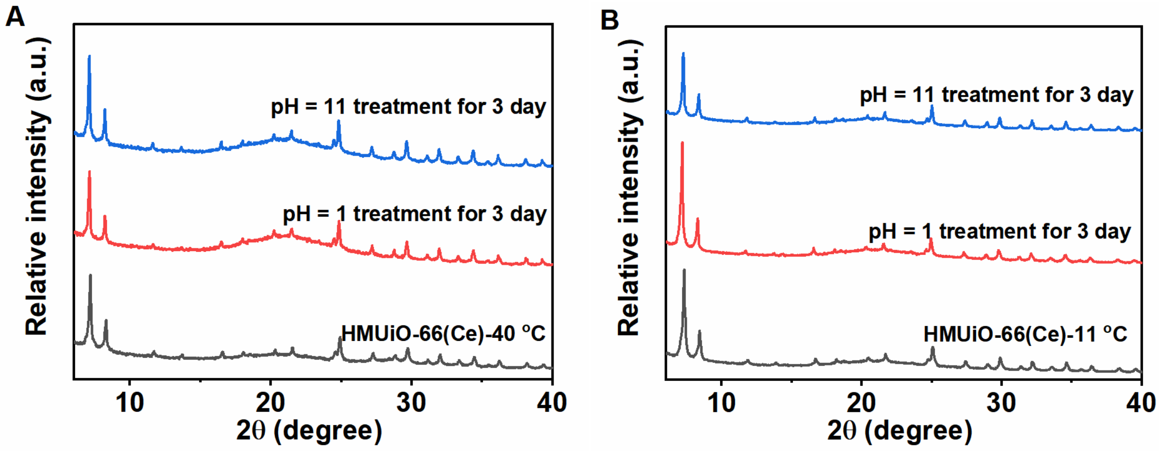

Figure S7. XRD patterns for HMUiO-66(Ce) samples synthesized at 40 °C and 11 °C after being soaked in (A) acidic and (B) alkaline aqueous solutions for 3 days at room temperature.

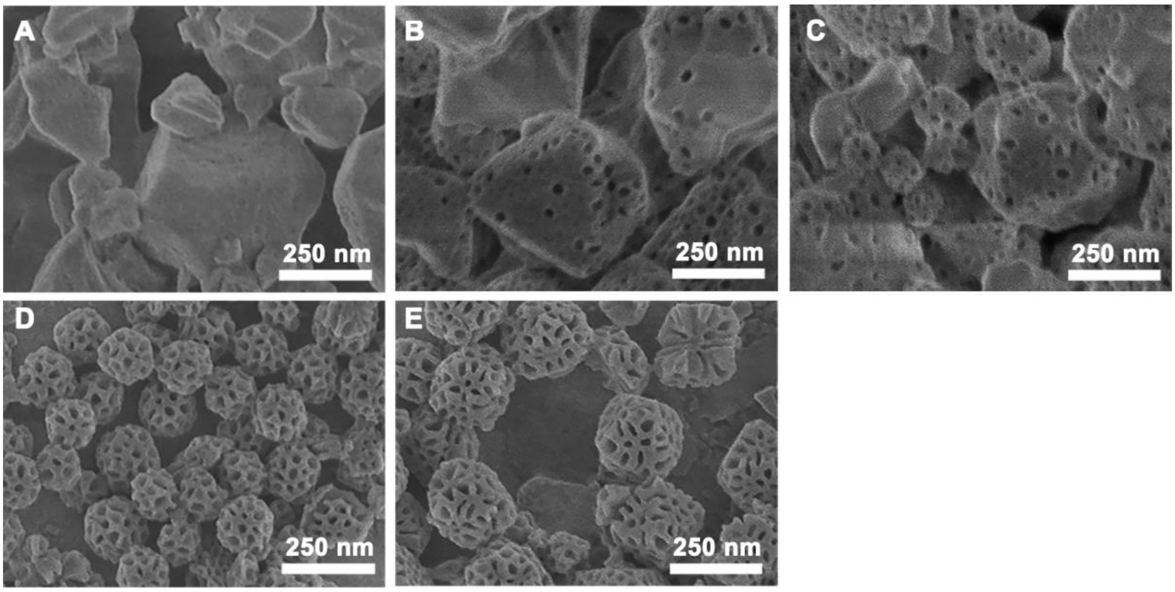

Figure S8. (A-E) SEM images of HMUiO-66(Ce) samples synthesized at 11 ^{o}C utilizing toluene/F127 microemulsions as templates. The toluene/F127 molar ratios were set as (A) 0, (B) 57, (C) 114, (D) 228, and (E) 455, respectively.

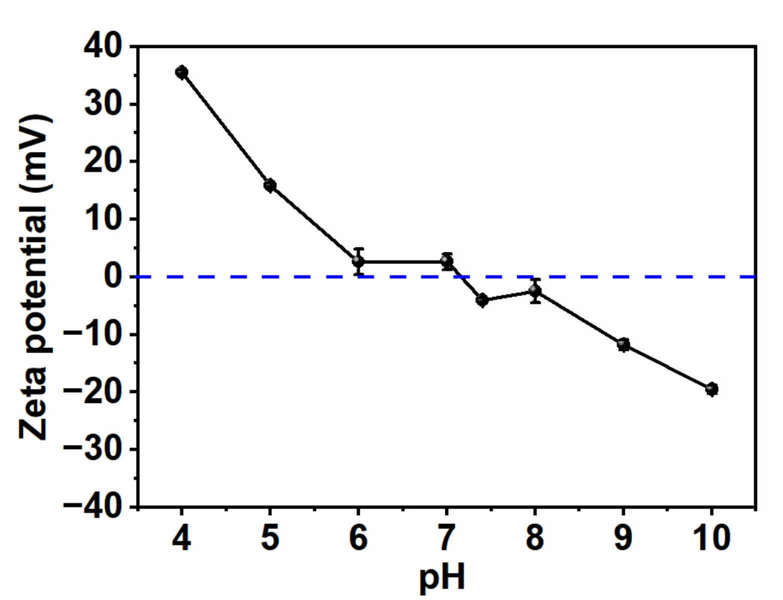

Figure S9. The zeta potential of HMUiO-66(Ce) under various pHs.

Limit of Detection for ALP with HMUiO-66(Ce)

Detection Limit = 3σ/slope

= 3 × 0.00333/(2.4×10^{-4})

= 42 fM

Multiple number of absorbance value at 570 nm (n = 5) were recorded for the blank sample of HMUiO-66(Ce). Sample standard deviation (σ) for the blank sample, without the addition of ALP was calculated to be 0.00333. The slope value was taken from a calibration curve for absorbance intensity against ALP concentration, which was calculated to be 2.4×10^{-4} (**Figure S10**).

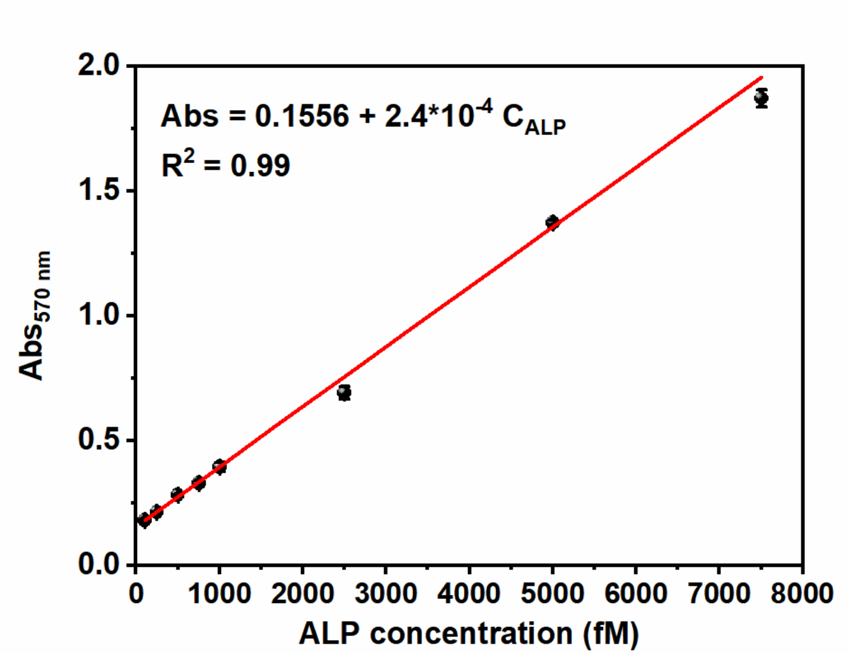

Figure S10. Relation of absorbance intensity against ALP concentration based on HMUiO-66(Ce) sensor and their linear fitting curve for the estimation of LOD.

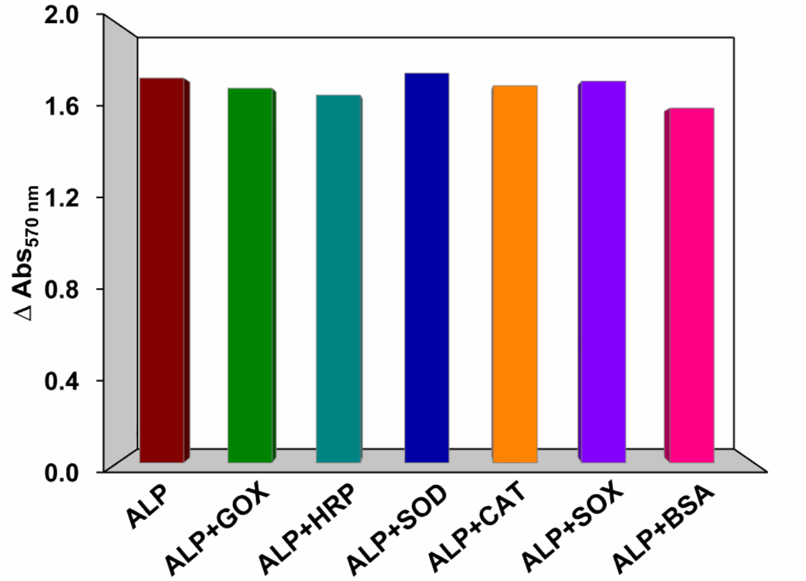

Figure S11. Absorbance response of HMUiO-66(Ce) towards ALP (7500 fM) in the coexistence of various interfering species (7500 fM) based on the cycling amplification strategy.

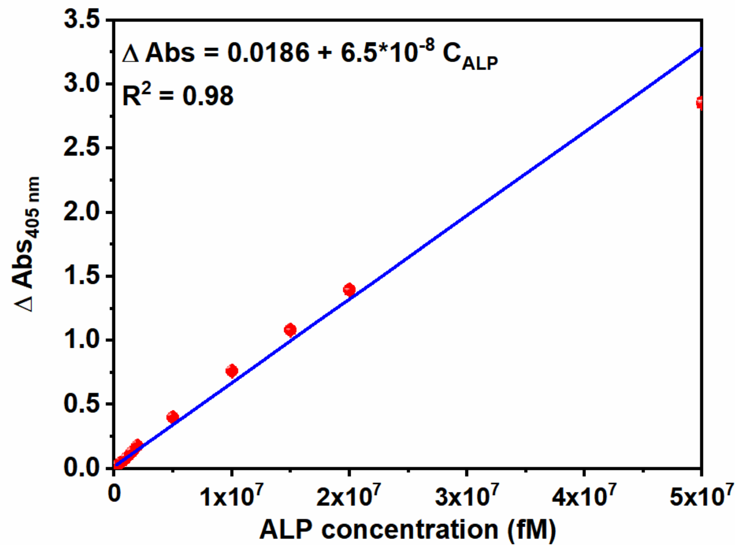

Figure S12. The absorbance change at 405 nm plotted against ALP concentrations using the classic colorimetric assay kit with PNPP method, along with the corresponding linear fitting curve. The ALP concentration was set as 2×10^5 fM, 5×10^5 fM, 1×10^6 fM, 1.5×10^6 fM, 2×10^6 fM, 5×10^6 fM, 1×10^7 fM, 1.5×10^7 fM, 2×10^7 fM and 5×10^7 fM, respectively.

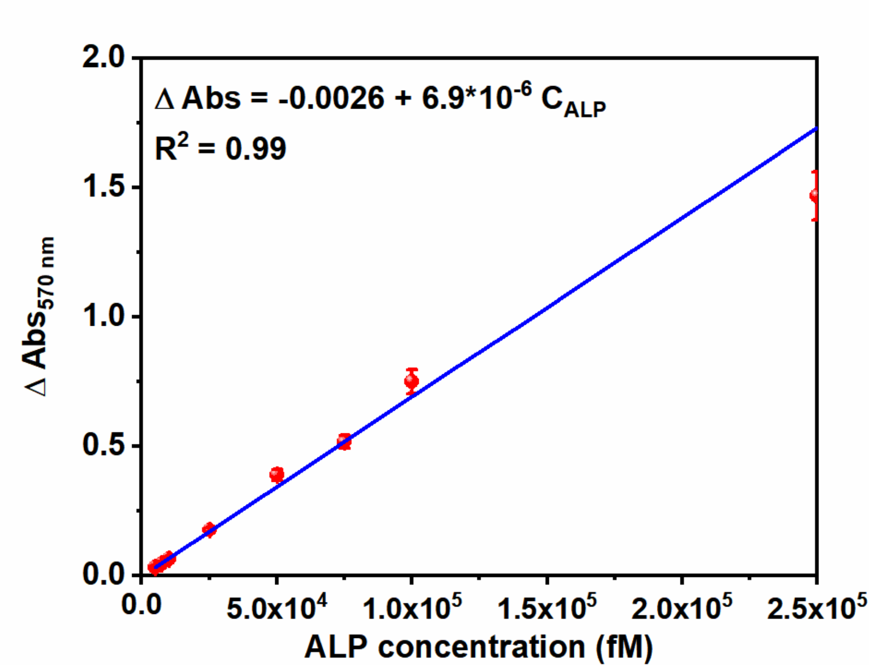

Figure S13. The absorbance change at 570 nm plotted against ALP concentrations using single cyclic amplification method without HMMOFs, along with the corresponding linear fitting curve. The ALP concentration was set as 5×10^3 fM, 7.5×10^3 fM, 1×10^4 fM, 2.5×10^4 fM, 5×10^4 fM, 7.5×10^4 fM, 1×10^5 fM and 2.5×10^5 fM, respectively.

Limit of Detection for ALP using Single Cyclic Amplification Method without HMMOFs

Detection Limit = 3σ/slope

= 3 × 0.00591/(6.9×10^{-6})

= 2571 fM

Multiple number of absorbance value at 570 nm (n = 5) were recorded for the blank sample. Sample standard deviation (σ) for the blank sample, without the addition of ALP was calculated to be 0.00591. The slope value was taken from a calibration curve for absorbance intensity against ALP concentration, which was calculated to be 6.9×10^{-6} (**Figure S14**).

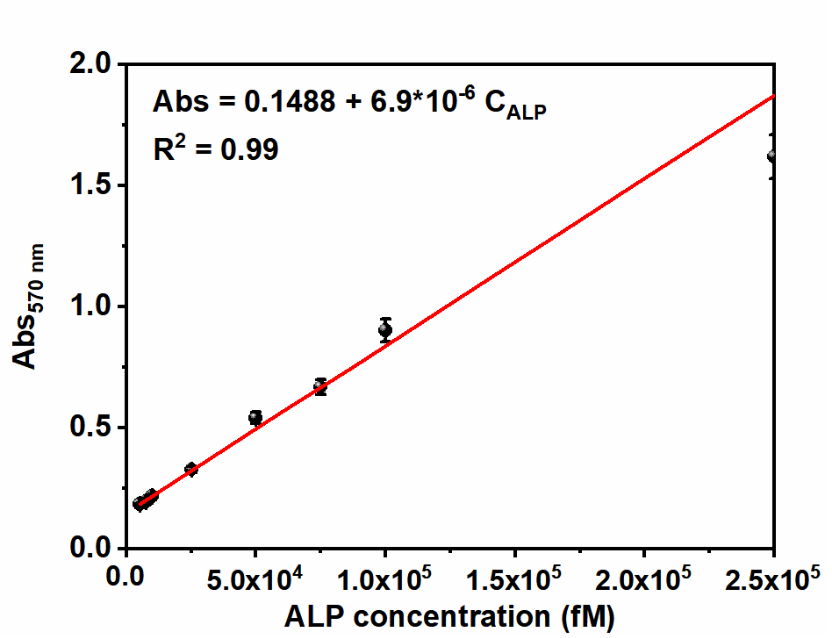

Figure S14. Relation of absorbance intensity against ALP concentration using single cyclic amplification method without HMMOFs and their linear fitting curve for the estimation of LOD.

Limit of Detection for ALP with Classic Colorimetric Assessment Kit based on PNPP Method

Detection Limit = 3σ/slope

= 3 × 0.00403/(6.5×10^{-8})

= 185777 fM

Multiple number of absorbance value at 405 nm (n = 5) were recorded for the blank sample. Sample standard deviation (σ) for the blank sample, without the addition of ALP was calculated to be 0.00403. The slope value was taken from a calibration curve for absorbance intensity against ALP concentration, which was calculated to be 6.5×10^{-8} (**Figure S15**).

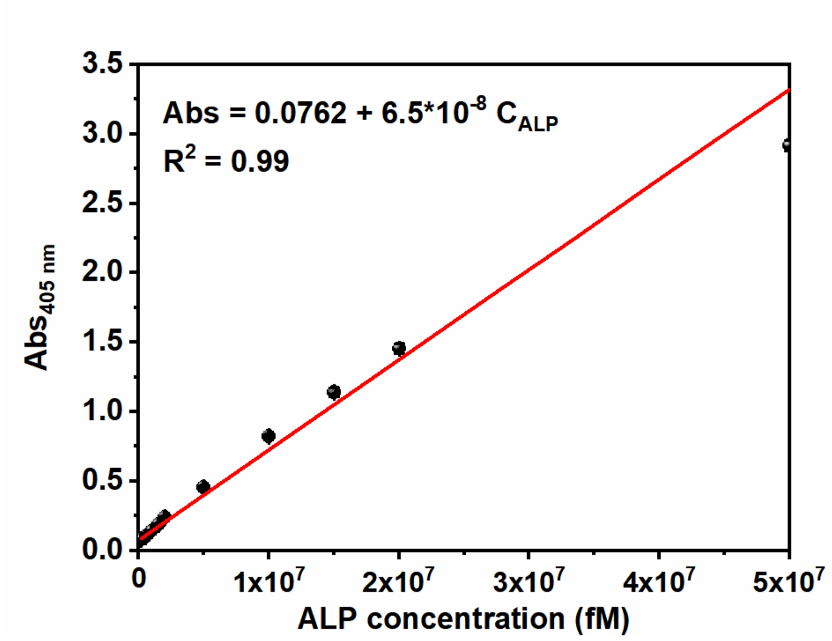

Figure S15. Relation of absorbance intensity against ALP concentration using the classic colorimetric assay kit with PNPP method and their linear fitting curve for the estimation of LOD.

Table S4. Comparison of the performance for ALP activity detection based on the recent developed colorimetric and fluorometric methods.

Methods	Sensory materials	Detection range (U/L)[a]	Fitting relationship	LOD (U/L)[a]	Refs.
Colorimetric	Au nanorods	5-100	Linear	3.3	[1]
Colorimetric	Ag NPs	0.15-5	Linear	0.037	[2]
Colorimetric	CoOOH nanoflflakes	0.04-160	Linear	0.026	[3]
Colorimetric	Ceria NPs	0.075-2	Linear	0.04	[4]
Colorimetric	PB nanocubes	0.6-6	Linear	0.23	[5]
Colorimetric	AuNRs	0.01-0.4	Linear	0.01	[6]
Colorimetric	Cu^{2+} and HRP	0-120	Linear	5.4	[7]
Colorimetric	$Cu(BCDS)_2{}^{2-}$	0-220	Non-linear	1.27	[8]
Colorimetric	Fe/C NS	0.05-6.00	Linear	0.03	[9]
Colorimetric	Cu-MOFs	1-34	Linear	0.19	[10]
Colorimetric	PDA nano-liposomes	0.01–0.20	Linear	0.0028	[11]
Colorimetric	Fe-N/C	0.05-100	Linear	0.02	[12]
Colorimetric	Cu@PB NCs	0.1-50	Linear	0.08	[13]
Fluorometric	CdTe QDs	1-1000	Linear	0.25	[14]
Fluorometric	ssDNA-templated AgNCs	1-100	Linear	0.63	[15]
Fluorometric	CDs and MnO_2 nanosheets	1-100	Linear	0.4	[16]
Fluorometric	AuNCs and $KMnO_4$	0.01-450	Linear	0.002	[17]
Fluorometric	F-PDA and MnO_2 nanosheets	1-80	Linear	0.34	[18]
Fluorometric	PDA nanodots	1-50	Linear	0.94	[19]
Fluorometric	NGQDs and CoOOH nanosheets	0.1-5	Linear	0.07	[20]
Fluorometric	Ascorbic acid 2-phosphate	1–100	Linear	0.50	[21]
Fluorescent	Gold nanocluster	0.47–10.0	Linear	0.09	[22]
Fluorometric	2,3-Diaminonaphthalene and AA	0.1–60	Linear	0.08	[23]
Colorimetric	HMUiO-66(Ce)	0.000327-0.0245	linear	0.00014	This work

[a]The activity of ALP used in this work is 38000 U/g, and we uniformly converted the concentration unit (fM) into enzyme activity unit (U/L) for comparison with the detection range and detection limit

between different literatures.

The activity unit (U/L) = (C_{ALP} × M_{ALP})* 38000

C_{ALP} is the concentration unit of ALP (M); M_{ALP} is the relative molecular weight of ALP, which is around 86000.[24]

Detection of ALP Activity Concentration in Human Serum Samples using the Commercialized ALP Detection Kit

The human serum samples, provided by Renji Hospital, Shanghai Jiaotong University School of Medicine, were collected from four volunteers. We detected the ALP activity concentration in human serum samples according to the instructions of the commercial ALP detection kit. The kit contains detection buffer, colorimetric substrate of PNPP solution, p-nitrophenol standard solution (10 mM), and reaction termination solution. The ALP activity concentration is defined as 1 U/L, which is the amount of alkaline phosphatase needed to hydrolyze 1 μM of the PNPP colorimetric substrate to produce 1 μM p-nitrophenol per min in a diethanolamine buffer of pH 9.8 at 37 ^{o}C.

First, we conducted a standard curve test for p-nitrophenol, which is the hydrolysis product of PNPP. Specifically, 10 μL of the p-nitrophenol standard solution (10 mM) was diluted with the detection buffer to obtain a p-nitrophenol solution with a concentration of 0.5 mM. Subsequently, it was further diluted to different concentrations of p-nitrophenol solution (0 μM, 20 μM, 40 μM, 80 μM, 120 μM, 160 μM, 200 μM). Then, 100 μL of the above p-nitrophenol solutions was added to a 96-well plate, followed by the addition of 100 μL of reaction termination solution. The absorbance at 405 nm was measured using a microplate reader. The enhanced absorbance values were obtained by subtracting the absorbance of the blank sample from the absorbance of p-nitrophenol samples at different concentrations. These enhanced absorbance were linearly fitted against the different p-nitrophenol concentrations to

establish the standard curve for p-nitrophenol, the hydrolysis product of PNPP. Finally, a linear fit was performed on these enhanced absorbance values versus the p-nitrophenol concentrations, generating the standard curve for the p-nitrophenol (**Figure S16**).

Then, ALP detection in human serum was carried out utilizing the commercial ALP detection kit. Each serum sample was assayed in four parallel setups. In the 96-well plate, 50 μL of detection buffer and 50 μL of colorimetric substrate solution were mixed as the blank control groups. Additionally, 10 μL of serum sample was added to a mixture of 40 μL detection buffer and 50 μL colorimetric substrate solution, resulting in 10-fold diluted serum samples. After the colorimetric reaction at 37 °C for 10 min, 100 μL of reaction termination solution was added to both the blank control groups and the serum sample groups. Absorbance at 405 nm was measured using a microplate reader after the colorimetric reaction. The enhanced absorbance values were obtained by subtracting the absorbance of the blank sample groups from the absorbance of serum sample groups. According to the p-nitrophenol standard curve, the concentrations of produced p-nitrophenol were calculated using the following formula (1):

$$C_{p-nitrophenol} = \frac{\Delta_{Abs} - 0.0039}{0.00555} \quad (1)$$

Where Δ_{Abs} represents the enhanced absorbance values at 405 nm for serum sample groups after the colorimetric reaction.

Finally, the activity concentrations of serum samples were calculated using the following formula (2):

$$C_{ALP}\ (U/L) = \frac{C_{p-nitrophenol}}{t} \times N \quad (2)$$

Where $C_{p-nitrophenol}$ (μM) represents the concentration of p-nitrophenol produced by each serum sample after the colorimetric reaction within 10 min, t (10 min) represents the colorimetric

reaction time, and N (10-fold) represents the dilution fold of the serum sample.

Detection of ALP Activity Concentration in Human Serum Samples using the Developed Method

First, we prepared standard solutions with different ALP concentrations (0 fM, 100 fM, 250 fM, 500 fM, 750 fM, 1000 fM, 2500 fM, 5000 fM), corresponding to ALP activity concentrations of 0 U/L, 0.0003268 U/L, 0.000817 U/L, 0.001634 U/L, 0.002451 U/L, 0.003268 U/L, 0.00817 U/L, and 0.01634 U/L. The ALP activity concentration standard curve was tested using these different ALP standard solutions (**Figure S17**), based on the standard colorimetric detection procedure of ALP in the presence of HMUiO-66(Ce). The notable variation was the use of a higher concentration of HMUiO-66(Ce) to meet the requirements for enriching ALP in serum samples, where 50 μL of HMUiO-66(Ce) suspension (250 mg/L, in ethanol) was added to the bottom of each tube.

Then, ALP in human serum was detected by using the developed method. Four parallel samples were set up for each human serum sample. The human serum sample was diluted 10^5-fold with Tris-HCl buffer (20 mM, pH 7.4), and then the standard colorimetric detection procedure of ALP in the presence of HMUiO-66(Ce) was conducted. Absorbance at 570 nm was measured using a microplate reader after the colorimetric reaction. The enhanced absorbance of the serum sample group was obtained by subtracting the absorbance of the blank sample groups from the absorbance of serum sample groups. Based on the standard curve of ALP activity concentration, the ALP activity concentration in human serum was calculated using the following formula (3):

$$C_{ALP}\ (U/L) = \frac{\Delta_{Abs} + 0.00683}{95.3} \times N \qquad (3)$$

Where Δ_{Abs} represents the enhanced absorbance values at 570 nm for serum sample groups after the colorimetric reaction, and N (10^5-fold) represents the dilution fold of the serum sample.

The spiked recovery tests were conducted by additional adding three different ALP concentrations (100 U/L, 300 U/L and 600 U/L) into the serum sample 1, in which already contains 105.3 U/L of ALP as determined by the commercialized ALP detection kit. Then, the total ALP concentration was measured from the prepared spiked samples in four parallel setups based on the developed method. The recovery was calculated (Recovery = detected concentration of total ALP/actual concentration of total ALP × 100).

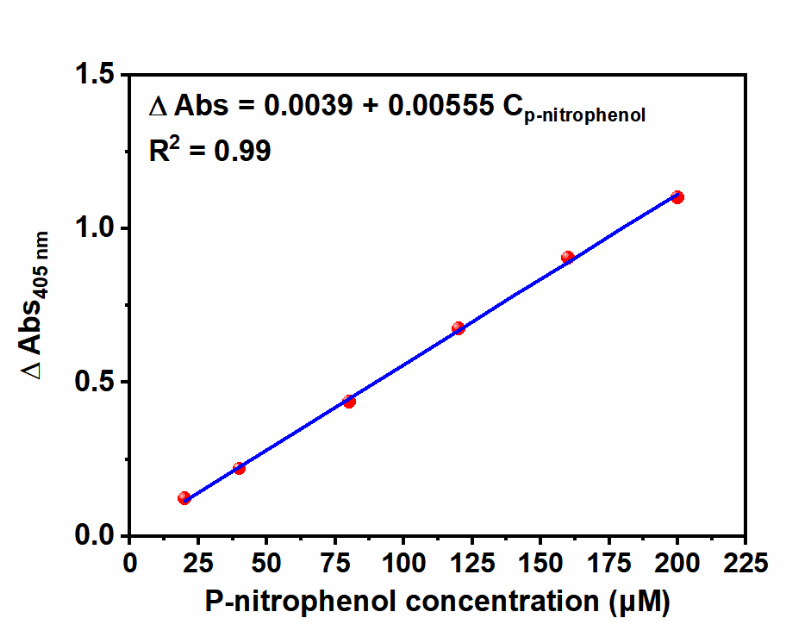

Figure S16. The fitting standard curve for the enhanced absorbance values against p-nitrophenol molar concentrations.

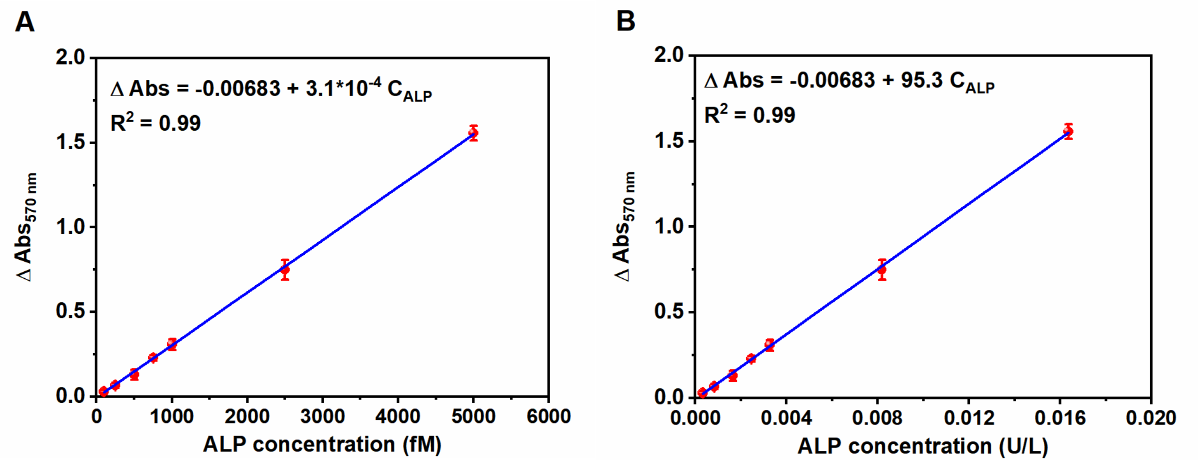

Figure S17. The fitting standard curve for the enhanced absorbance values against (A) ALP molar concentrations and (B) ALP activity concentrations. Higher concentrations of HMUiO-66(Ce) (50 μL of 250 mg/L HMUiO-66(Ce)) were used in the developed method to meet the requirements of enriching ALP in serum samples.

Table S5. The comparison of detected ALP in human serum samples from four volunteers by using the commercialized ALP detection kit and our developed method.

Sample number	Detected ALP (U/L) by commercialized kit	RSD (%, n=4)	Detected ALP (U/L) by developed method	RSD (%, n=4)
**Serum 1**	105.3 ± 8.0	7.6	103.3 ± 6.6	5.7
**Serum 2**	99.4 ± 5.7	5.7	99.1 ± 8.8	6.4
**Serum 3**	114.8 ± 6.0	5.2	110.4 ± 8.2	7.4
**Serum 4**	117.1 ± 9.8	8.3	120.7 ± 11.7	9.7

Table S6. Recovery of the additional added ALP in serum samples determined by the developed method.

Already contained ALP in serum (U/L)	Additional added ALP (U/L)	Total ALP (U/L)	Detected ALP (U/L) by developed method	Recovery (%)	RSD (%, n=4)
	100	205.3	195.6 ± 11.1	95.3	5.7
105.3[a]	300	405.3	375.9 ± 19.8	92.8	5.1
	600	705.3	719.9 ± 15.6	102.1	2.2

[a] *The serum sample 1 was used to added with extra ALP, in which already contains 105.3 U/L of ALP as determined by the commercialized ALP detection kit.*

References

1. Z. Gao, K. Deng, X,-D. Wang, M. Miró, D. Tang, *ACS Appl. Mater. Interfaces*. **2014**, *6*, 18243.
2. H. Song, Z. Li, Y. Peng, X. Li, X. Xu, J. Pan, X. Niu, *Analyst*. **2019**, *144*, 2416.
3. S. G. Liu, L. Han, N. Li, N. Xiao, Y. J. Ju, N. B. Li, H. Q. Luo, *J. Mater. Chem. B* **2018**, *6*, 2843.
4. A. Hayat, G. Bulbul S. Andreescu, *Biosens. Bioelectron*. **2014**, *56*, 334.
5. T. Wu, W. Hou, Z. Ma, M. Liu, X. Liu, Y. Zhang, S. Yao, *Microchim. Acta* **2019**, *186*, 123.
6. Z. Zhang, Z. Chen, S. Wang, F. Cheng, L. Chen, *ACS Appl. Mater. Interfaces* **2015**, *7*, 27639.
7. D. Shi, Y. Sun, L. Lin, C. Shi, G. Wang, J. Zhang, *Analyst* **2016**, *141*, 5549.
8. Q. Hu, B. Zhou, F. Li, J. Kong, X. Zhang, *Chem. Asian J* **2016**, *11*, 3040.
9. X. Zhou, M. Wang, M. Wang, X. Su, *ACS Appl. Nano Mater*. **2021**, *4*, 7888.
10. C. Wang, J. Gao, Y. Cao, H. Tan, *Anal. Chim. Acta* **2018**, *1104*, 74.
11. D. Wang, S. You, W. Huo, X. Han, H. Xu, *Microchim. Acta* **2022**, *189*, 70.
12. Q. Chen, S. Li, Y. Liu, X. Zhang, Y. Tang, H. Chai, Y. Huang, *Sens. Actuators B Chem*. **2020**, *305*, 127511.
13. S. Fan, X. Jiang, M. Yang, X. Wang, *Anal. Bioanal. Chem.* **2021**, *413*, 3955.
14. P. Chen, S. Yan E. Sawyer, B. Ying, X. Wei, Z. Wu, J. Geng, *Analyst* **2019**, *44*, 1147.
15. Y. He, B. Jiao, *Microchim. Acta* **2017**, *184*, 4167.
16. F. Qu, H. Pei, R. Kong, S. Zhu, L. Xia, *Talanta* **2017**, *165*, 136.

17. X. Hu, X. Wu, X. Fang, Z. Li, G. Wang, *Biosens. Bioelectron.* **2016**, *77*, 666.

18. T. Xiao, J. Sun, J. Zhao, S. Wang, G. Liu, X. Yang, *ACS Appl. Mater. Interfaces* **2018**, *10*, 6560.

19. Q. Xue, X. Cao, C. Zhang, Y. Xian, *Microchim. Acta* **2018**, *185*, 231.

20. J. Liu, D. Tang, Z. Chen, X. Yan, Z. Zhong, L. Kang, J. Yao, *Biosens. Bioelectron.* **2017**, *94*, 271.

21. X. Zhou, F. Khusbu, H. Chen, C. Ma, *Talanta* **2023**, *208*, 120453.

22. L. Liu, H. Jiang, X. Wang, *Biosens. Bioelectron.* **2021**, *173*, 112786.

23. J. Wen, Y. Hu, N. Li, D. Li, G. Zheng, Y. Zou, M. Zhang, L. Shui, *Anal. Chim. Acta* **2022,** *1230*, 340414.

24. J. A. Reynolds, M. J. Schlesinger, *Biochemistry* **1969**, *8*, 588.
